# Pulsatile Intravascular Lithotripsy for Heavily Calcified Femoropopliteal Arterial Disease: First-in-Human Results from the POWER PAD I Feasibility Study

**DOI:** 10.3390/jcm15176589

**Published:** 2026-08-26

**Authors:** Bibombe Patrice Mwipatayi, Bernardette Jingfei Lee, Davina Daudu, Nelson Encarnación Santana, Alexandra Lansky, Robert S. Chisena, Hitinder S. Gurm, Robert Traficante, Jon C. George

**Affiliations:** 1 School of Surgery, University of Western Australia, Perth, WA 6000, Australia; 2 Department of Vascular and Endovascular Surgery, Sir Charles Gairdner Hospital, Perth, WA 6009, Australia; 3 Department of Vascular and Endovascular Surgery, Royal Perth Hospital, WA 6009, Australia; 4 Department of Vascular and Endovascular Surgery, Centro Medico Moderno, Santo Domingo 10132, Dominican Republic; drnencarnacion@gmail.com; 5 Yale School of Medicine, Yale University, New Haven, CT 06510, USA; 6 AVS, Inc., Waltham, MA 02451, USA; robert.chisena@avspulse.com; 7 Division of Cardiovascular Medicine, Department of Internal Medicine, Frankel Cardiovascular Center, University of Michigan, Arbor, MI 48109, USA; 8 Statistical Revelations Pty Ltd., Sandringham, VIC 3191, Australia; robert@statisticalrevelations.com.au; 9 Department of Interventional Cardiology and Endovascular Medicine, Pennsylvania Hospital, University of Pennsylvania Health System, Philadelphia, PA 19104, USA

**Keywords:** peripheral arterial disease, femoropopliteal artery, arterial calcification, pulsatile intravascular lithotripsy, lesion preparation, endovascular intervention, calcium modification, feasibility study

## Abstract

**Background/Objectives:** Severe arterial calcification remains a major challenge during femoropopliteal endovascular intervention, limiting luminal gain and increasing the risk of vessel-wall injury, restenosis, and bailout stenting. Pulsatile intravascular lithotripsy (PIVL) is a novel calcium-modification technology that converts pneumatic pulses into short-duration hydraulic pressure waves delivered through a non-compliant balloon. POWER PAD I evaluated the first-in-human feasibility, safety, and procedural performance of PIVL in calcified femoropopliteal arterial disease. **Methods:** POWER PAD I was a prospective, single-arm, two-center feasibility study. Adults with Rutherford category 2–4 symptomatic peripheral arterial disease and moderately or severely calcified superficial femoral or popliteal lesions were treated with PIVL, followed by adjunctive therapy when clinically indicated. Angiographic and duplex ultrasound outcomes were assessed by an independent core laboratory, and adverse events were adjudicated by an independent clinical events committee. **Results:** Nine patients underwent treatment of 20 lesions. Mean age was 76.2 ± 12.6 years, 19 lesions were severely calcified, and five lesions were chronic total occlusions. Patient-level device and procedural success were each achieved in 8 of 9 patients; technical success was achieved in all patients. Mean diameter stenosis decreased from 76.5 ± 18.0% before treatment to 28.1 ± 6.9% after PIVL-containing lesion preparation and before adjunctive drug-coated-balloon angioplasty, and to 20.6 ± 5.8% after adjunctive therapy. Mean minimal luminal diameter increased from 1.3 ± 1.0 mm to 3.8 ± 0.5 mm after PIVL-containing lesion preparation and to 4.3 ± 0.6 mm at final angiography. No perforation, distal embolization, thrombus, abrupt closure, no-reflow, bailout stenting, or grade D or higher dissection occurred. Freedom from clinically driven target-lesion revascularization was 100% at 30 days and 6 months. **Conclusions:** PIVL-containing lesion preparation was feasible in heavily calcified superficial femoral and popliteal lesions and was associated with substantial acute luminal gain before adjunctive DCB therapy and no severe angiographic complications. Favorable 6-month clinical and patency outcomes were observed after the combined treatment strategy of PIVL vessel preparation and adjunctive DCB therapy; therefore, longer-term outcomes cannot be attributed to PIVL alone. Larger controlled studies are warranted.

## 1. Introduction

Peripheral arterial disease (PAD) affects more than 200 million people worldwide and is associated with impaired walking capacity, reduced quality of life, recurrent hospitalization, major cardiovascular events, limb loss, and premature mortality [1,2,3,4]. Symptomatic femoropopliteal disease accounts for a substantial proportion of lower-extremity PAD and is commonly treated with endovascular revascularization. Although contemporary endovascular therapy has improved procedural success and reduced the need for open surgical reconstruction in appropriately selected patients, durability remains strongly influenced by lesion morphology [5,6].

Among the anatomic factors that complicate peripheral vascular intervention, arterial calcification is particularly important. Calcification reduces vessel elasticity and compliance, limits balloon expansion, impairs luminal gain, compromises the delivery and effectiveness of drug-coated balloons and drug-eluting technologies, and increases the risk of recoil, dissection, perforation, restenosis, and repeat revascularization [7,8,9,10,11,12]. These challenges are especially relevant in the superficial femoral and popliteal arteries, where lesions are frequently long, eccentric, circumferentially calcified, or chronically occluded. These arteries are also exposed to flexion, compression, torsion, and elongation, which makes adequate lesion preparation and the avoidance of permanent implants clinically important.

Several strategies have been developed to address calcified femoropopliteal disease. High-pressure angioplasty may improve the luminal diameter but can increase vessel wall injury. Scoring and cutting balloons may focus dilatation forces but remain limited by ability to cross the lesion and complexity. Atherectomy can debulk or modify calcified plaque, but adds procedural complexity, cost, and a potential risk of distal embolization or perforation [13,14,15,16]. These limitations have led to increasing interest in intravascular lithotripsy (IVL), which modifies calcium through dynamic impulse energy rather than plaque excision or static dilatation.

Electrohydraulic IVL has represented an important advance in the treatment of calcified coronary and peripheral arterial disease [17,18,19,20,21,22,23,24]. In these systems, emitters mounted within a semi-compliant balloon generate ultrasonic pressure waves that propagate through the balloon inflation medium to disrupt intimal and medial calcium. Clinical studies and real-world experience have supported the procedural feasibility and early safety of electrohydraulic IVL in calcified peripheral lesions, particularly as a vessel-preparation strategy before definitive angioplasty or drug delivery [19,20,21,22,23,24].

Pulsatile intravascular lithotripsy (PIVL; Pulse IVL System, AVS, Inc., Waltham, MA, USA) is a novel IVL platform with a distinct mechanism of action. Rather than generating electrohydraulic pulses from emitters within the balloon, the system induces the “water hammer effect” by converting pneumatic carbon dioxide pulses into hydraulic pressure waves, accelerating those waves through a waveguide, and transmitting the pressure waves through the incompressible saline-contrast medium in the catheter shaft. These cyclical pressure waves are distributed through the balloon and directed towards the vessel wall, with the intent of modifying arterial calcium while maintaining low average balloon pressure. Preclinical studies have shown that PIVL can generate full-thickness fractures in intimal, medial, circumferential, and eccentric calcium; improve vessel compliance; and facilitate luminal expansion without requiring a separate high-pressure dilatation step [25].

These preclinical findings provided the rationale for POWER PAD I, a first-in-human feasibility study designed to evaluate the safety and performance of PIVL in calcified superficial femoral and popliteal arterial disease. Initial acute angiographic outcomes for the patients and lesions enrolled in POWER PAD I were previously reported in a research letter in *JACC: Cardiovascular Interventions* [26]. The present manuscript reports the same study population and includes those previously reported acute core laboratory-assessed angiographic outcomes, while substantially expanding the analysis to include detailed baseline and procedural characteristics and angiographic, duplex ultrasound, clinical, and safety outcomes from POWER PAD I.

## 2. Materials and Methods

### 2.1. Study Design and Oversight

POWER PAD I was a first-in-human, prospective, single-arm, two-center feasibility study evaluating the Pulse Peripheral Intravascular Lithotripsy Balloon Catheter in patients with symptomatic calcified superficial femoral and popliteal arterial disease. The study was conducted at Royal Perth Hospital, Perth, Western Australia, and Centro Medico Moderno, Santo Domingo, Dominican Republic.

The study was registered at ClinicalTrials.gov (NCT05192473) and conducted in accordance with the Declaration of Helsinki, ISO 14155:2020, Good Clinical Practice, and applicable regulatory requirements [27]. The protocol was approved by the relevant local ethics committee or institutional review board at each participating center. Written informed consent was obtained from all participants before enrolment.

**Figure 1:**
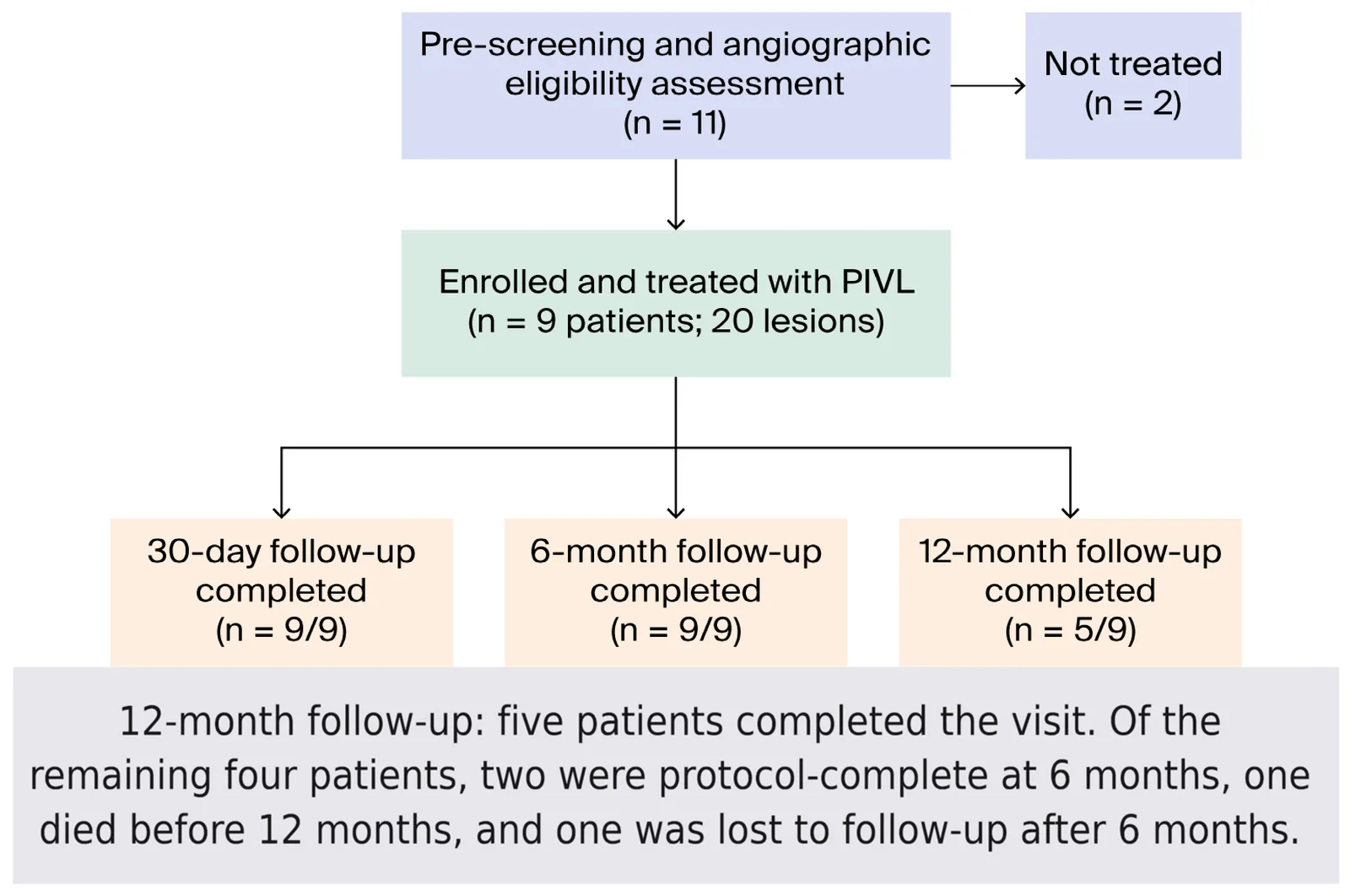
POWER PAD I Study Flow. Patients underwent pre-screening and angiographic eligibility assessment. Of the 11 patients assessed, 9 were enrolled and treated with pulsatile intravascular lithotripsy, involving 20 lesions. All treated patients completed 30-day and 6-month follow-up. Five patients completed 12-month follow-up; of the remaining 4 patients, 2 were protocol-complete at 6 months, 1 died before 12 months, and 1 was lost to follow-up after 6 months. PIVL denotes pulsatile intravascular lithotripsy.

POWER PAD I was conducted as a prospective, interventional, single-arm, non-randomized, first-in-human feasibility study. Reporting was guided by the TREND (Transparent Reporting of Evaluations with Nonrandomized Designs) statement for non-randomized interventional studies. A completed TREND-based reporting checklist is provided as Supplementary Table S1, and patient disposition, treatment, and follow-up are summarized in Figure 1.

### 2.2. Patients

Patients were eligible if they were 18 years of age or older and had symptomatic lower-extremity PAD involving site-reported, calcified stenotic lesions of the superficial femoral and/or popliteal arteries. Three lesions were classified by the independent core laboratory as common femoral arteries and were retained in the analysis with explicit lesion-level reporting. Eligible patients had Rutherford category 2, 3, or 4 disease and objective evidence of arterial insufficiency, defined as a resting ABI of 0.90 or less, or an exercise ABI of 0.75 or less.

Angiographic eligibility required at least one and up to five distinct target lesions separated by a segment of angiographically healthy vessel with a minimum length of 2 cm and not requiring treatment; target-lesion diameter stenosis greater than 50%; a reference vessel diameter between 4.0 and 6.5 mm; an individual lesion length of 150 mm or less; a total treatment length less than 350 mm; and at least moderate calcification according to PARC criteria [28]. Successful guidewire crossing through the true lumen, without flow-limiting dissection or perforation, was required before treatment. At least one patent tibial runoff vessel to the ankle was also required.

The key exclusion criteria included Rutherford category 5 or 6 disease, mild calcification only, target lesions involving grafts or in-stent restenosis, severe tortuosity precluding safe device delivery, chronic total occlusion longer than 40 mm, aneurysm, visible thrombus, and any lesion considered unsuitable for safe endovascular treatment. One treated patient had a sponsor-approved protocol deviation after core-laboratory review and showed only 1 cm of healthy tissue between lesions rather than the protocol-required minimum of 2 cm of angiographically healthy tissue between lesions. This patient was retained in the analysis to preserve the complete first-in-human treated cohort.

### 2.3. Device Description and Interventional Procedure

The Pulse IVL System consists of a console, handle, and non-compliant balloon catheter delivered over an exchange-length 0.014-inch guidewire. The balloon is primed with saline-contrast medium at approximately 1 atm. When treatment is initiated, pneumatic carbon dioxide pulses generated by the console and handle are converted by an amplifier into hydraulic pulses in the balloon inflation medium. A diaphragm within the amplifier propagates longitudinal, acoustic waves through the catheter and into the balloon at approximately 15 pulses per second.

The resulting pressure waves radiate through the inflation medium to the target vessel wall. The system is designed to exert a transient peak impact pulse of approximately 50 atm over a short time, while maintaining a low average balloon pressure of approximately 6–8 atm. This mechanism is intended to fracture intraluminal calcium and facilitate luminal expansion while minimizing barotrauma. The system is intended for PIVL-enhanced balloon dilatation of calcified and fibro-calcific lesions in the peripheral vasculature and is not intended for coronary or cerebral use.

Procedures were performed by trained operators using standard endovascular techniques. After angiographic confirmation of eligibility, the PIVL catheter was positioned across each target lesion, and therapy was delivered according to the investigational device instructions. A minimum treatment duration of 30 s per target lesion was recommended.

Angiographic imaging was obtained before treatment, immediately after PIVL and before adjunctive therapy, and after the completion of adjunctive therapy. Adjunctive therapy, including drug-coated balloon (DCB) angioplasty, intravascular ultrasound, embolic protection, or other clinically indicated measures, was permitted at the operators’ discretion. The DCB platform was not protocol-mandated, and manufacturer/model information was not consistently captured in the study dataset. Bailout stenting was permitted if required but was not used in any lesion.

### 2.4. End Points and Definitions

The primary performance outcomes were device success, technical success, and procedural success. Device success was defined at the patient level as successful delivery, balloon inflation, balloon deflation, and retrieval of the study device without balloon rupture prior to the rated lifespan of the catheter. Technical success was defined as successful vascular access, completion of the endovascular procedure with or without adjunctive therapy, and the immediate achievement of residual stenosis of 50% or less by quantitative angiography. Procedural success was defined as the achievement of device success and technical success in the absence of procedural complications.

Secondary performance outcomes included freedom from CD-TLR, achievement of final residual stenosis less than 30%, change in Rutherford category, change in ABI, change in EQ-5D-5L visual analogue scale score, change in WIQ scores, and duplex ultrasound patency. Duplex ultrasound patency was defined as the absence of restenosis of 50% or greater, assessed by PSVR criteria and core-laboratory interpretation. Patient-level primary patency was described as freedom from CD-TLR together with the absence of restenosis of 50% or greater in the treated lesion set.

The primary safety end point was the rate of major adverse limb events at 30 days. Major adverse limb events were defined as major unplanned amputation of the target limb or major reintervention. The secondary safety end point was the rate of major adverse events at 30 days, including major adverse limb events and procedure-related death. Additional safety outcomes included all serious adverse events, adverse events adjudicated as related to the device or procedure, all-cause death, and late restenosis events.

### 2.5. Follow-Up

Clinical assessments were performed at discharge, 30 days, 6 months, and, where required by protocol, 12 months after the index procedure. Duplex ultrasound assessments were scheduled at 30 days, 6 months, and 12 months when applicable.

### 2.6. Statistical Analysis

POWER PAD I was designed as a first-in-human feasibility study to evaluate the procedural performance, early safety, and angiographic effect of PIVL in calcified superficial femoral and popliteal arterial disease. The study was not powered to test a formal superiority, non-inferiority, or comparative-effectiveness hypothesis. All analyses were therefore descriptive and exploratory, with emphasis on procedural feasibility, independent core-laboratory findings, follow-up completeness, and concordance between angiographic, duplex ultrasound, clinical, and safety outcomes.

The primary analysis population included all patients who underwent attempted treatment with the study device. Outcomes are reported according to the relevant unit of analysis. The patient-level outcomes included baseline clinical characteristics, device success, technical success, procedural success, Rutherford category, ABI, patient-reported outcomes, CD-TLR, major adverse limb events, major adverse events, death, and adverse events. The lesion-level outcomes included calcification severity, lesion length, diameter stenosis, MLD, dissection, perforation, distal embolization, thrombus, abrupt closure, no-reflow, bailout stenting, and duplex ultrasound patency. The device-level outcomes included balloon delivery, inflation, deflation, rupture, and retrieval. This distinction was maintained to avoid overstating precision in a small study in which several patients contributed more than one lesion.

Continuous variables are summarized as mean ± standard deviation, median with range, or both, as appropriate. Categorical variables are summarized as counts and percentages, with denominators shown explicitly when data were incomplete or when outcomes were reported at the patient, lesion, or device level. Descriptive confidence intervals were reported for protocol-defined feasibility end points where available; given the small sample size, these intervals were interpreted as measures of statistical imprecision rather than confirmatory evidence.

Angiographic outcomes were assessed by an independent core laboratory before PIVL, immediately after PIVL and before adjunctive therapy, and after adjunctive therapy when performed. Absolute and relative changes in percentage diameter stenosis and MLD were calculated from these serial measurements. The change from baseline to post-PIVL was compared with the total change from baseline to final angiography to describe the immediate contribution of PIVL separately from the incremental effect of adjunctive therapy.

Because multiple lesions could be treated within the same patient, lesion-level angiographic and duplex ultrasound results were interpreted with awareness of within-patient clustering and are presented descriptively rather than as independent confirmatory observations. No imputation of missing data was performed. Analyses were based on observed data, with evaluable denominators reported at each follow-up time point. Thirty-day and 6-month outcomes were considered the principal follow-up findings because all treated patients completed these visits. Twelve-month outcomes were considered exploratory because follow-up was available in five patients only.

Confidence limits are presented at the 95% level. To facilitate conventional clinical interpretation, exact 95% confidence intervals for the principal feasibility outcomes are provided in Supplementary Table S2. No *p* values were used to support efficacy claims. All statistical analyses were performed using SAS software, version 9.4 (SAS Institute Inc., Cary, NC, USA). Further information regarding the mixed model statistical analysis is provided in the Supplemental Material.

## 3. Results

### 3.1. Patient Disposition and Follow-Up Completeness

Between July 2022 and May 2023, 11 patients met pre-screening criteria and underwent angiographic assessment for eligibility. Nine patients were enrolled and treated with PIVL. Seven patients were treated at Royal Perth Hospital, Australia, and two patients were treated at Centro Medico Moderno, Dominican Republic. All nine treated patients were included in the effectiveness and safety analyses (Figure 1).

Follow-up was completed over 6 months. All nine patients completed 30-day and 6-month assessments. Five patients completed 12-month follow-up. Of the remaining four patients, two were protocol-complete at 6 months, one died before the 12-month visit, and one was lost to follow-up after 6 months. Therefore, 30-day and 6-month outcomes represent complete follow-up of the treated population, whereas 12-month findings are reported descriptively in the evaluable subset.

### 3.2. Baseline Clinical and Lesion Characteristics

The median age of treated patients was 80 years, with a range of 60 to 94 years; the mean age was 76.2 ± 12.6 years. Five patients were male. Two thirds were white, and six patients were current or former smokers. Five patients reported heavy tobacco exposure. Hypertension was present in eight patients with available data, coronary heart disease in six patients, previous myocardial infarction in two patients, and diabetes requiring treatment in two patients. At baseline, two patients had Rutherford category 2 disease, four had Rutherford category 3 disease, and three had Rutherford category 4 disease.

A total of 20 lesions were treated in nine patients, corresponding to a mean of 2.2 lesions per patient. Although all lesions were reported by the investigational sites as being within the protocol-permitted superficial femoral and popliteal anatomy at the time of enrollment, independent core laboratory adjudication subsequently classified fifteen lesions as being located in the superficial femoral artery, three in the common femoral artery, and two in the above-knee popliteal artery. The lesion cohort was highly calcified and anatomically complex: 19 of 20 lesions met the PARC criteria for severe calcification, one lesion was moderately calcified, and five lesions were chronic total occlusions. Calcium morphology (i.e., nodular, eccentric, concentric) was not a prespecified angiographic variable and was not systematically adjudicated via core lab analysis.

The mean reference vessel diameter was 5.2 ± 1.0 mm. The mean lesion length was 75 ± 36 mm per lesion and 166 ± 120 mm per patient. The mean calcified length was 88 ± 40 mm per lesion and 195 ± 144 mm per patient. The mean PIVL treatment length was 96 ± 40 mm per lesion and 212 ± 129 mm per patient. Pretreatment angiographic severity was substantial, with mean baseline diameter stenosis of 76.5 ± 18.0% and a mean pretreatment MLD of 1.3 ± 1.0 mm. The baseline characteristics are summarized in Table 1.

### 3.3. Procedural Characteristics and Device Performance

Femoral arterial access was obtained successfully in all patients. For the five chronic total occlusions, guidewire crossing was achieved through the true lumen by angiographic assessment before PIVL; no subintimal wire course, flow-limiting dissection, or perforation was reported. Intravascular imaging was not systematically performed to further characterize wire position.

The mean procedure duration was 115 ± 39 min, with a median duration of 124 min. The mean fluoroscopy time was 26 ± 12 min, and mean contrast volume was 135 ± 44 mL. Fifteen PIVL balloons were used to treat 20 lesions. A median of two devices was used per patient, with a range of one to four devices.

Pre-dilatation was performed in 11 of 20 lesions, embolic protection was used in three patients, and adjunctive DCB angioplasty was performed after PIVL in 19 of 20 lesions. No lesion required bailout stent implantation. The DCB platform was selected at operator discretion and was not consistently captured in the core study dataset; therefore, no DCB-platform-specific analysis was possible. The frequent use of DCB angioplasty is important when interpreting longer-term patency. Serial angiography was performed before treatment, immediately after PIVL and before adjunctive DCB therapy, and after adjunctive therapy; however, because a separate post-pre-dilatation core-laboratory angiographic assessment was not prespecified, the change from baseline angiography to the immediate post-PIVL/pre-DCB angiogram should be interpreted as the angiographic effect observed after PIVL-containing lesion preparation before adjunctive DCB therapy. Given the severe calcific burden of the treated lesions, this improvement is most consistent with PIVL-mediated calcium modification, although the isolated contribution of pre-dilatation cannot be formally quantified.

At the patient level, device success was achieved in 8 of 9 patients (88.9%; 95% CI, 51.8–99.7%). The single device-success failure was due to balloon rupture prior to the catheter’s rated life of 5000 pulses. The rupture occurred after therapy had been delivered as intended (approximately 3600 pulses or 4 min of treatment); the system detected the failure and shut off automatically. There were no associated clinical sequelae.

Technical success was achieved in all nine patients (100%; 95% CI, 66.4–100%). In each case, vascular access was successful, the endovascular procedure was completed, and final residual stenosis was 50% or less. Procedural success, defined as device success and technical success without procedural complications, was achieved in 8 of 9 patients (88.9%; 95% CI, 51.8–99.7%). At the device level, all 15 balloons were successfully delivered, inflated, deflated, and retrieved, with rupture-free performance in 14 of 15 devices.

### 3.4. Core-Laboratory Angiographic Outcomes and Vessel Wall Safety

Independent core-laboratory angiography demonstrated substantial acute luminal improvement after PIVL. Mean percentage diameter stenosis decreased from 76.5 ± 18.0% before treatment to 28.1 ± 6.9% immediately after PIVL. This represented an absolute reduction of 48.5 percentage points before adjunctive therapy and a relative reduction of approximately 64%. After adjunctive therapy, mean residual stenosis decreased further to 20.6 ± 5.8%, corresponding to an overall absolute reduction of 55.9 percentage points from baseline and a relative reduction of approximately 73%. A sensitivity analysis excluding common femoral lesions is included in the Supplementary Information.

**Figure 2:**
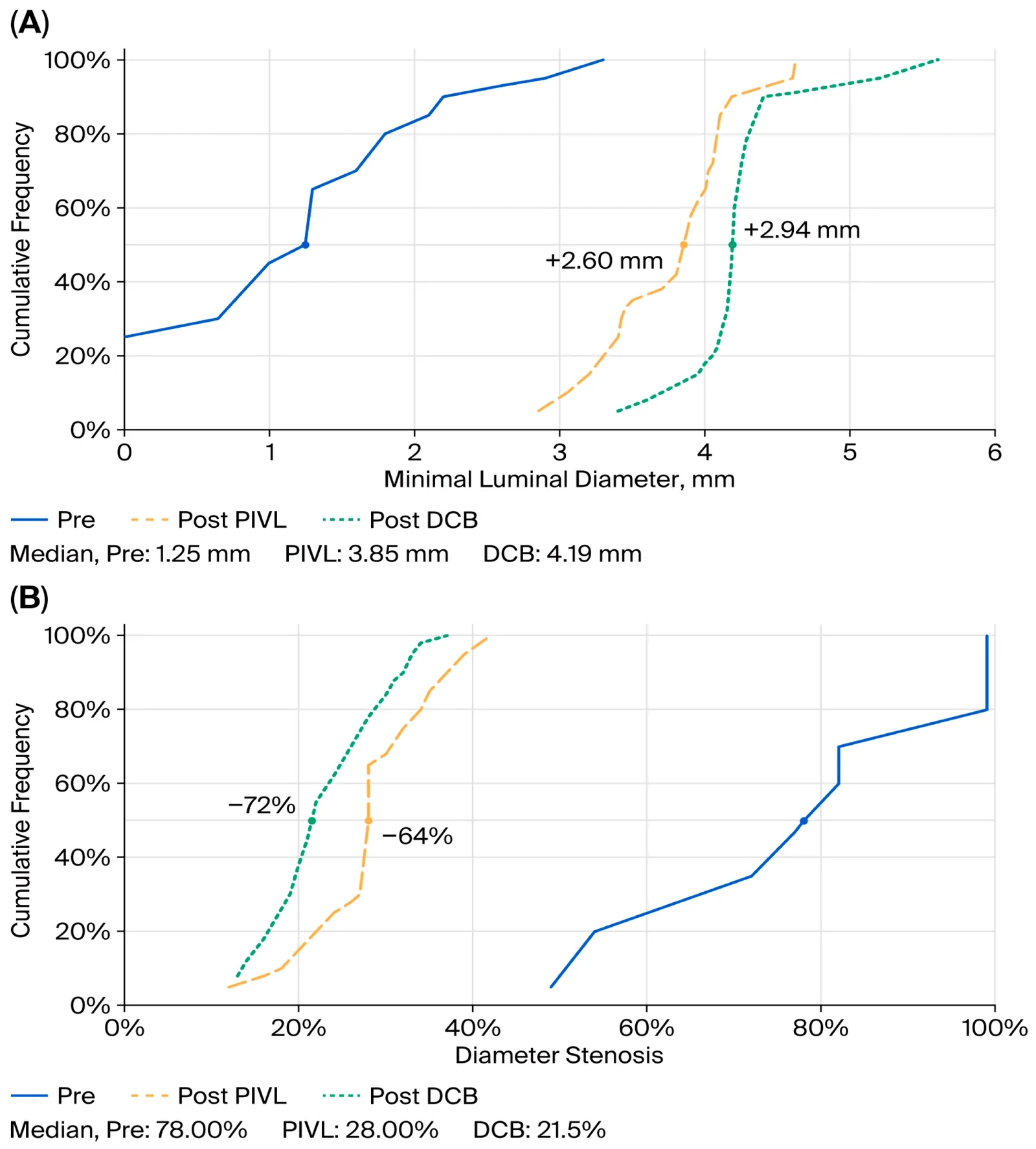
Core laboratory angiographic cumulative frequency distributions. Cumulative frequency distributions of minimal luminal diameter (**A**) and diameter stenosis (**B**) at baseline, after PIVL and before adjunctive drug-coated-balloon angioplasty, and after adjunctive drug-coated-balloon angioplasty. Median minimal luminal diameter increased from 1.25 mm at baseline to 3.85 mm after PIVL and 4.19 mm after DCB angioplasty, corresponding to absolute gains of +2.60 mm and +2.94 mm, respectively. Median diameter stenosis decreased from 78.00% at baseline to 28.00% after PIVL and 21.50% after DCB angioplasty, corresponding to relative reductions of 64% and 72%, respectively. DCB denotes drug-coated balloon; PIVL, pulsatile intravascular lithotripsy.

In the descriptive mixed-model analysis, the estimated absolute reduction in residual stenosis was 48.5 percentage points after PIVL (95% CI, 45.6 to 51.4 percentage points) and 55.9 percentage points after completion of all treatment steps (95% CI, 53.1 to 58.8 percentage points). These intervals are reported descriptively and should not be interpreted as confirmatory inference. Cumulative frequency distributions of MLD and percentage diameter stenosis are shown in Figure 2.

Most of the final angiographic improvement occurred during the PIVL step. Approximately 85% of the total reduction in residual stenosis was observed between pretreatment angiography and post-PIVL angiography, before adjunctive treatment. The MLD changed in parallel. The mean MLD increased from 1.3 ± 1.0 mm at baseline to 3.8 ± 0.5 mm immediately after PIVL, corresponding to an acute luminal gain of 2.5 ± 1.1 mm. The final mean MLD after adjunctive therapy was 4.3 ± 0.6 mm. Approximately 84% of the total increase in luminal diameter occurred during the PIVL step.

**Figure 3:**
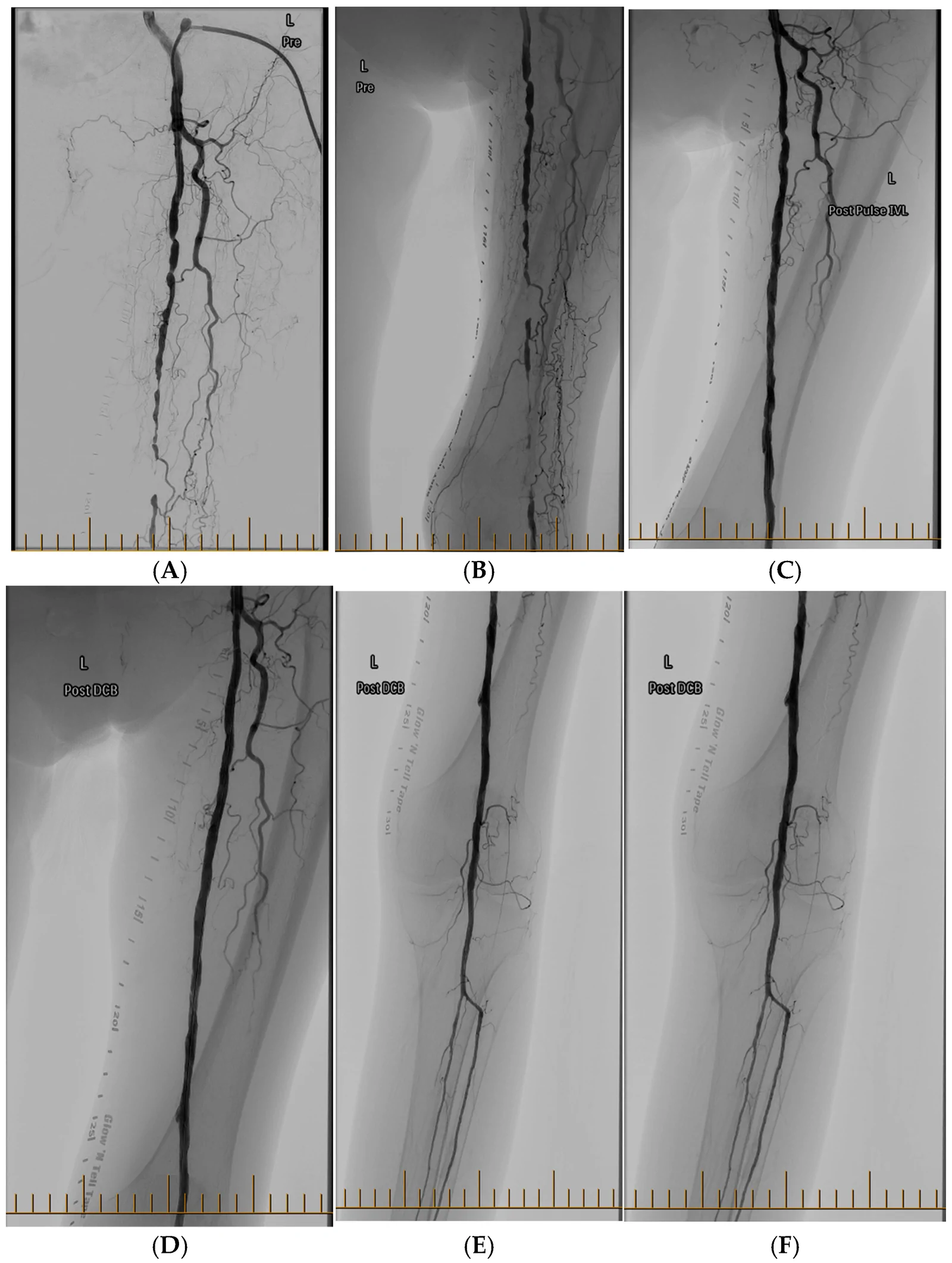
Procedural angiographic. Representative procedural angiographic images from a 94-year-old woman with Rutherford category 4 peripheral arterial disease and four calcified target lesions involving the superficial femoral and popliteal arteries. Panels (**A**–**C**) show the proximal superficial femoral artery at baseline, after PIVL-containing lesion preparation and before adjunctive drug-coated-balloon angioplasty, and after adjunctive drug-coated-balloon angioplasty, respectively. Panels (**D**–**F**) show the distal superficial femoral and popliteal segments at the corresponding procedural time points. Horizontal reference markers indicate comparable anatomical levels and treated segments across the image series. No bailout stent was implanted, and all treated lesions remained patent with less than 50% restenosis at 6 months. DCB denotes drug-coated balloon; PIVL, pulsatile intravascular lithotripsy.

All 20 lesions achieved final residual stenosis of 50% or less, and 18 of 20 lesions achieved final residual stenosis of 30% or less. No lesion developed angiographic perforation, thrombus, distal embolization, abrupt closure, no-reflow, or grade D or higher dissection. Dissection was absent in 15 lesions, grade B in three lesions, and grade C in two lesions. A representative angiographic example is shown in Figure 3, and procedural and angiographic outcomes are summarized in Table 2.

### 3.5. Duplex Ultrasound Patency, Clinical Outcomes, and Safety

Freedom from CD-TLR was 100% at both 30 days and 6 months. One CD-TLR occurred by 12 months. Duplex ultrasound patency, defined as absence of restenosis of 50% or greater by PSVR criteria, was maintained in 20 of 20 lesions at 30 days and 19 of 20 lesions at 6 months. Patient-level primary patency, defined as freedom from CD-TLR with maintained duplex patency in the treated lesion set, was 9 of 9 patients (100%) at 30 days and 8 of 9 patients (88.9%) at 6 months. The 6-month duplex ultrasound findings are the most robust data because all treated patients completed this visit; 12-month findings are exploratory because only five patients completed 12-month follow-up.

**Figure 4:**
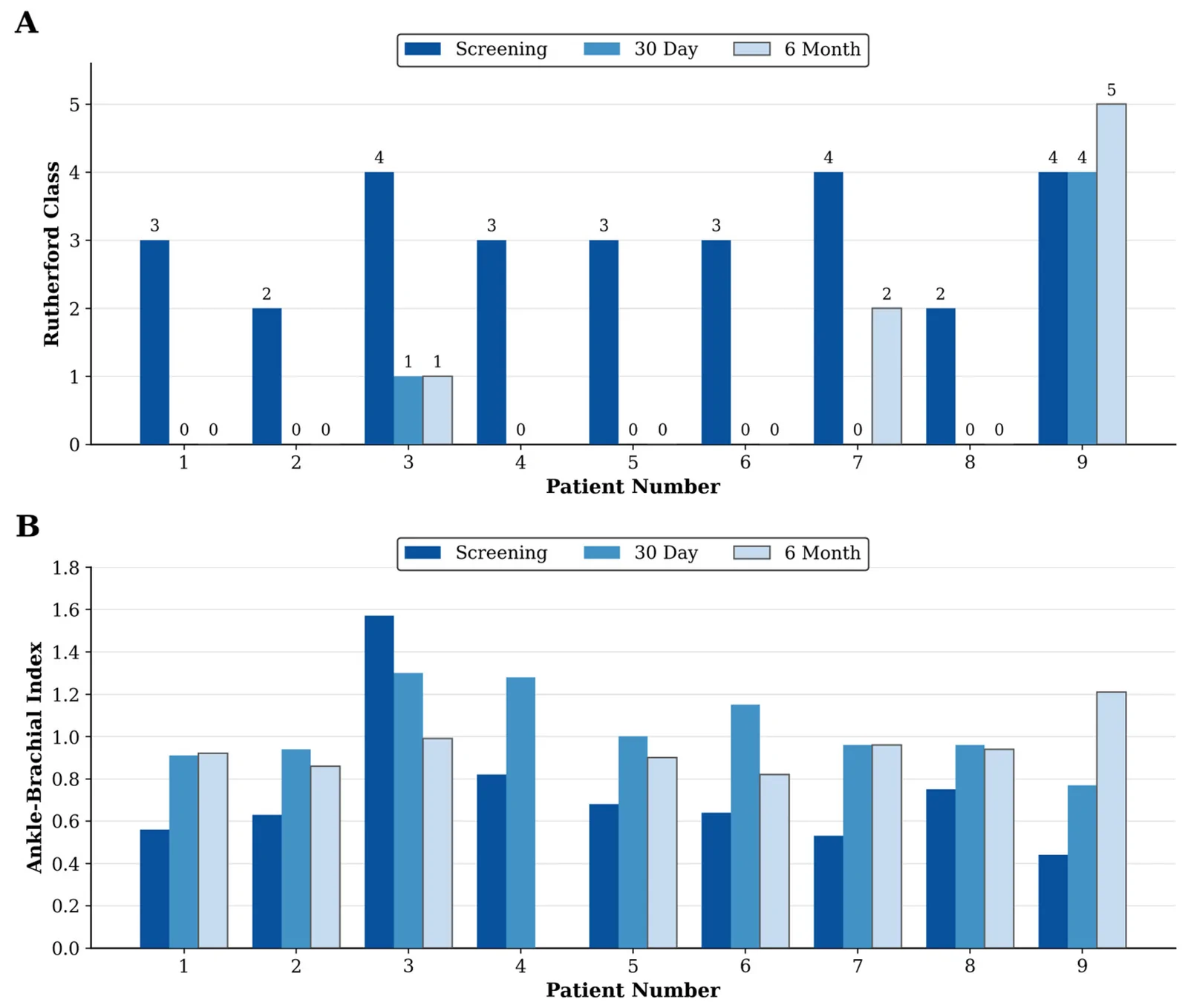
Clinical and hemodynamic outcomes in the treated limb. Patient-level changes in Rutherford category and ankle–brachial index from screening to 30 days and 6 months after PIVL-containing lesion preparation are shown. Panel (**A**) shows the Rutherford category for each treated patient. Panel (**B**) shows the ankle–brachial index for each treated limb. Small baseline squares in Panel (**A**) indicate Rutherford category 0. ABI denotes ankle–brachial index; PIVL, pulsatile intravascular lithotripsy.

Clinical improvement was consistent with the angiographic and duplex ultrasound findings. At screening, 78% of patients had Rutherford category 3 or 4 disease. Rutherford category improvement was observed in 8 of 9 patients at 30 days and in 88% of evaluable patients at 6 months. Among patients with PAD by baseline ABI criteria, the ABI improved in all evaluable patients at both 30 days and 6 months (Figure 4).

Patient-reported health status also improved. The mean EQ-5D-5L visual analogue scale score increased from 47.5 ± 17.8 at screening to 78.8 ± 17.1 at 30 days and 73.6 ± 24.3 at 6 months. Among patients with available 12-month data, the mean score was 69.0 ± 16.7. WIQ domains improved from baseline, with the greatest mean improvements observed in walking impairment and walking distance.

There were no adjudicated adverse events meeting definitions for major unplanned amputation of the target limb, major reintervention, major adverse limb event, procedure-related death, or major adverse event at 30 days. No procedure-related death occurred during follow-up. Across the full follow-up period, 15 serious adverse events occurred in five patients. None was adjudicated as device related. One serious adverse event, an access site hematoma that began immediately after the index procedure, was considered related to the procedure. Other serious adverse events were clinically heterogeneous and included cardiac failure, pulmonary edema, gastrointestinal bleeding, atrial fibrillation, urinary retention, peripheral ischemia, and non-device-related clinical events.

One patient died of cardiac failure 303 days after the index procedure. The death was not considered related to the study device or procedure. Five non-serious device- or procedure-related adverse events occurred before discharge in four patients, including vascular access dissection or access-site complications, extremity pain, and arterial restenosis. Three device-related adverse events occurred between 6 and 12 months in two patients; all were peripheral arterial restenosis events. Follow-up, clinical outcomes, and safety findings are summarized in Table 3.

### 3.6. Integrated Interpretation of Results

The results demonstrate a coherent feasibility signal. The device was deliverable and retrievable in all devices use, technical success was achieved in all patients, acute luminal gain was substantial by independent core-laboratory angiography, and no bailout stenting was required. Severe angiographic complications were not observed. Much of the final reduction in stenosis and increase in MLD occurred during the PIVL step, before adjunctive therapy, supporting the biological plausibility of lithotripsy-mediated calcium modification and luminal expansion.

Clinical and duplex ultrasound outcomes through 6 months were directionally consistent with the acute angiographic findings. However, because POWER PAD I was a small, single-arm, first-in-human study and adjunctive DCB angioplasty was used in most lesions, the findings should be interpreted as hypothesis-generating rather than definitive evidence of comparative effectiveness or long-term durability. The analysis of populations, denominators, and outcome units are summarized in Table 4.

## 4. Discussion

In this first-in-human feasibility study, pulsatile intravascular lithotripsy (PIVL) was feasible for the treatment of heavily calcified superficial femoral and popliteal arterial disease and was associated with substantial acute luminal gain, no bailout stenting, no severe angiographic vessel wall complications, and favorable 6-month clinical and duplex ultrasound outcomes. The treated lesions were anatomically complex: 95% were severely calcified, 25% were chronic total occlusions, and several patients had multiple target lesions. These features are clinically important because arterial calcification reduces vessel compliance, limits balloon expansion, increases the risk of recoil and dissection, and is associated with restenosis and repeat revascularization after peripheral endovascular intervention [7,8].

The principal finding was that most of the angiographic improvement occurred immediately after PIVL and before adjunctive therapy. Mean percentage diameter stenosis decreased from 76.5% at baseline to 28.1% at the immediate post-PIVL assessment, and the mean minimal luminal diameter increased from 1.3 mm to 3.8 mm. The final post-adjunctive result was a residual stenosis of 20.6% and a minimal luminal diameter of 4.3 mm. Approximately 85% of the total reduction in residual stenosis and 84% of the total increase in minimal luminal diameter occurred during the PIVL step. These findings suggest that PIVL produced meaningful acute lesion preparation and luminal expansion before adjunctive drug-coated-balloon angioplasty.

Although most of the final angiographic improvement was observed before adjunctive DCB angioplasty, pre-dilatation was performed in 11 lesions to facilitate lesion crossing and delivery of the PIVL catheter. A separate post-pre-dilatation core-laboratory angiographic assessment was not prespecified; therefore, the isolated contribution of pre-dilatation cannot be formally quantified. Nevertheless, pre-dilatation was used as a facilitative step rather than as definitive calcium-modification therapy. Given the severe calcific burden of the treated cohort, including 19 severely calcified lesions and five chronic total occlusions, the magnitude of acute luminal gain observed before adjunctive DCB angioplasty is most consistent with a clinically meaningful calcium-modification effect during PIVL-containing lesion preparation. Accordingly, the angiographic findings should be interpreted as reflecting the effect of a PIVL-containing lesion-preparation strategy before adjunctive therapy, rather than the effect of adjunctive DCB angioplasty or pre-dilatation alone.

Severely calcified superficial femoral and popliteal disease remains difficult to treat with conventional endovascular strategies. High-pressure balloon angioplasty may improve the luminal diameter but can result in uncontrolled vessel wall injury. Scoring and cutting balloons may focus dilatation forces, but their effectiveness may be limited in long, eccentric, circumferentially calcified, or occlusive lesions. Atherectomy can debulk or modify calcified plaque, but it increases procedural complexity and may be associated with distal embolization, perforation, and additional cost [13,14]. Restenosis after peripheral intervention is multifactorial and is influenced by recoil, dissection, neointimal hyperplasia, persistent calcific constraint, and impaired drug delivery, all of which may be amplified by severe calcification [15,16]. A vessel-preparation strategy that improves compliance while limiting barotrauma and severe dissection is therefore clinically attractive.

Intravascular lithotripsy has emerged as an important calcium-modification strategy in calcified coronary and peripheral arterial disease [17,18,19,20]. In peripheral arteries, the Shockwave electrohydraulic IVL program has provided the largest body of clinical evidence. The Disrupt PAD II and Disrupt PAD III studies showed that IVL could modify calcified femoropopliteal lesions and facilitate subsequent angioplasty with favorable acute procedural safety [22,24]. In the randomized DISRUPT PAD III trial, IVL used before drug-coated-balloon angioplasty improved procedural success compared with conventional balloon angioplasty in patients with moderately or severely calcified femoropopliteal lesions [22]. Mid-term DISRUPT PAD III follow-up further supported the durability of IVL-assisted vessel preparation through 1 and 2 years [29].

Observational data have extended this evidence base to broader and more complex peripheral arterial disease. The DISRUPT PAD III Observational Study included heavily calcified lesions treated in real-world practice, including infrapopliteal disease, and reported favorable early safety and procedural outcomes [21,30]. A recent 3-year single-center experience also supports the practical feasibility of IVL as an adjunctive vessel-preparation strategy in severe peripheral arterial calcification, particularly in complex femoropopliteal disease [31]. These studies provide an important clinical benchmark for POWER PAD I, but they do not permit direct comparison because the device platform, mechanism of action, study design, lesion mix, adjunctive therapy, and follow-up strategy differ.

The present study should therefore be interpreted within this evolving IVL landscape. Like Shockwave balloon-based IVL, PIVL is intended to modify arterial calcium through pressure-wave energy rather than plaque excision or high-pressure barotrauma. However, the technologies are not identical. Electrohydraulic IVL uses emitters within a balloon to generate ultrasonic pressure waves, whereas PIVL converts pneumatic pulses into hydraulic pressure waves within a saline-contrast medium contained in a non-compliant balloon. Preclinical work has shown that PIVL can fracture intimal and medial calcium, including eccentric and circumferential calcium, and can increase vessel compliance and luminal area [25]. The current first-in-human findings are consistent with this proposed mechanism, although calcium fracture was not directly confirmed because systematic intravascular imaging was not performed.

POWER PAD I also expands on the previously reported brief feasibility experience of pulse intravascular lithotripsy in calcified superficial femoral and popliteal arteries [26]. The present manuscript provides a fuller account of patient disposition, denominator-specific analysis, core-laboratory angiographic changes before and after adjunctive therapy, duplex ultrasound findings, clinical follow-up, and independently adjudicated safety outcomes. This distinction is important because early device studies can overstate the treatment effect if patient-level, lesion-level, and device-level outcomes are not clearly separated.

The FORWARD PAD experience is also relevant because it reflects the continued development of IVL technologies for complex calcified peripheral disease. The Shockwave Javelin Peripheral IVL catheter is a non-balloon, forward-facing IVL system designed to facilitate the treatment of severely narrowed or occlusive calcified lesions [32]. This platform differs from both conventional balloon-based electrohydraulic IVL and the non-compliant balloon-based PIVL system evaluated in POWER PAD I. Together, these studies show that IVL technology is expanding beyond a single device design, with different platforms intended to address different procedural challenges, including calcium modification, lesion crossing, vessel preparation, luminal expansion, and the avoidance of provisional stenting. However, no head-to-head comparison has been performed between PIVL, Shockwave balloon-based IVL, or forward-facing IVL systems; therefore, any comparison must remain mechanistic and contextual rather than inferential.

The angiographic safety findings in POWER PAD I were encouraging. No treated lesion developed perforation, distal embolization, thrombus, abrupt closure, no-reflow, or grade D or higher dissection, and no bailout stent was implanted. This is notable given the high burden of severe calcification and chronic total occlusion. The avoidance of provisional stenting may be particularly relevant in the femoropopliteal segment, where repetitive flexion, compression, elongation, and torsion can affect long-term implant durability. Nevertheless, the absence of severe angiographic complications in 20 lesions should be interpreted as an early feasibility signal rather than definitive evidence of safety.

The distinction between patient-level, lesion-level, and device-level outcomes is central to interpretation. Patient-level device success was achieved in 8 of 9 patients because one balloon ruptured prior to the rated lifespan of the catheter. At the device level, all 15 balloons were successfully delivered, inflated, deflated, and retrieved, and 14 of 15 were rupture-free. The balloon rupture occurred after therapy delivery, triggered automatic system shut off, and had no clinical sequelae. This event should be reported transparently. It does not negate the procedural feasibility observed in the study, but it identifies an important device-performance issue for continued engineering evaluation and larger clinical studies.

Clinical and duplex ultrasound outcomes were directionally consistent with the acute angiographic findings. All treated patients completed 30-day and 6-month follow-up. Freedom from clinically driven target-lesion revascularization was 100% at both time points, and duplex ultrasound demonstrated at least 50% patency in 19 of 20 lesions at 6 months. Rutherford category, ankle–brachial index, EQ-5D-5L visual analogue score, and Walking Impairment Questionnaire domains also improved among evaluable patients. These findings are encouraging but remain hypothesis-generating because the study was small and single-arm and used adjunctive DCB angioplasty in 19 of 20 lesions. One clinically driven target-lesion revascularization and three restenosis events occurred between 6 and 12 months, underscoring the need for longer-term surveillance.

This study has several limitations. First, POWER PAD I was a first-in-human feasibility study that enrolled only 9 patients with 20 treated lesions and was therefore not powered to provide precise estimates of safety, patency, restenosis, or reintervention. Second, the absence of a control group precludes conclusions regarding superiority or non-inferiority compared with conventional angioplasty, specialty balloons, atherectomy, Shockwave balloon-based IVL, or forward-facing IVL platforms. Third, adjunctive DCB angioplasty was performed in 19 of 20 lesions, limiting attribution of longer-term patency and clinical improvement to PIVL alone. In addition, the DCB platform was not protocol-mandated, and manufacturer/model information was not consistently captured, precluding assessment of whether DCB selection influenced follow-up outcomes. Fourth, although serial angiography allowed the immediate post-PIVL result to be distinguished from the final post-adjunctive result, systematic intravascular ultrasound, optical coherence tomography, or computed-tomography-based calcium assessment was not performed; consequently, calcium fracture, plaque modification, changes in vessel compliance, and effectiveness in various calcium morphologies could not be directly characterized in vivo. Fifth, 12-month follow-up was incomplete and should be considered exploratory. Finally, the study was sponsored by the device manufacturer, although independent core-laboratory assessment and independent clinical-event adjudication were used to strengthen objectivity and reduce assessment bias.

Despite these limitations, POWER PAD I provides first-in-human evidence that PIVL can be delivered in severely calcified superficial femoral and popliteal lesions with substantial acute luminal gain and without frequent severe angiographic complications. These findings support larger prospective studies with standardized adjunctive therapy, systematic intravascular imaging, longer follow-up, and appropriate comparators to define the safety, durability, mechanism of action, and comparative effectiveness of PIVL.

## 5. Conclusions

PIVL-containing lesion preparation was feasible in this first-in-human study of heavily calcified superficial femoral and popliteal arterial disease and was associated with substantial acute luminal gain before adjunctive DCB therapy, no bailout stenting, and no early adjudicated major adverse limb events or major adverse events. Given the frequent use of adjunctive DCB angioplasty, longer-term patency and clinical outcomes should be interpreted as reflecting a PIVL-containing treatment strategy rather than PIVL alone. Larger prospective studies are required to define the safety, mechanism, durability, and comparative effectiveness of PIVL.

## Figures and Tables

**Table 1:** Baseline patient and lesion characteristics.

Characteristic	Value
Patients treated	9
Age, years	76.2 ± 12.6; median 80 (range, 60–94)
Male sex	5/9 (55.6%)
Race	White: 6/9 (66.7%); Black or African American: 2/9 (22.2%); Asian: 1/9 (11.1%)
Current or former smoker	6/9 (66.7%)
Heavy tobacco use among smokers	5/6 (83.3%)
Hypertension	8/8 (100.0%)
Coronary heart disease	6/9 (66.7%)
Previous myocardial infarction	2/9 (22.2%)
Diabetes requiring treatment	2/9 (22.2%)
Baseline Rutherford category	Class 2: 2/9 (22.2%);
	Class 3: 4/9 (44.4%);
	Class 4: 3/9 (33.3%)
Lesions treated	20
Lesions per patient	Mean 2.2; median 2 (range, 1–4)
Lesion location	Superficial femoral artery: 15/20 (75.0%);
	Common femoral artery: 3/20 (15.0%);
	Above-knee popliteal artery: 2/20 (10.0%)
Severe calcification by PARC criteria	19/20 (95.0%)
Moderate calcification by PARC criteria	1/20 (5.0%)
Chronic total occlusion	5/20 (25.0%)
Reference vessel diameter, mm	5.2 ± 1.0
Lesion length, mm	75 ± 36 per lesion; 166 ± 120 per patient
Calcified length, mm	88 ± 40 per lesion; 195 ± 144 per patient
PIVL treatment length, mm	96 ± 40 per lesion; 212 ± 129 per patient
Baseline diameter stenosis, %	76.5 ± 18.0 (range, 46.7–100.0)
Baseline MLD, mm	1.3 ± 1.0

Values are presented as n/N (%), mean ± standard deviation, or median (range), or as otherwise stated. Patient-level variables use a denominator of nine treated patients unless otherwise indicated; lesion-level variables use a denominator of 20 treated lesions. MLD, minimal luminal diameter; PARC, Peripheral Academic Research Consortium; PIVL, pulsatile intravascular lithotripsy.

**Table 2:** Procedural, device, and angiographic outcomes.

Outcome	Value
Procedure duration, min	115 ± 39; median 124
Fluoroscopy time, min	26 ± 12
Contrast volume, mL	135 ± 44
Lesions treated per patient	Mean 2.2; median 2 (range, 1–4)
PIVL balloons used	15
Embolic protection	3/9 patients (33.3%)
Pre-dilatation	11/20 lesions (55.0%)
Post-PIVL DCB angioplasty	19/20 lesions (95.0%) *
Bailout stenting	0/20 lesions (0.0%)
Patient-level device success	8/9 (88.9%; 95% CI, 51.8–99.7%) **
Technical success	9/9 (100%; 95% CI, 66.4–100%)
Procedural success	8/9 (88.9%; 95% CI, 51.8–99.7%)
Successful balloon delivery, inflation, deflation, and retrieval	15/15 devices (100%)
Balloon rupture-free performance	14/15 devices (93.3%)
Diameter stenosis, %	Baseline: 76.5 ± 18.0;
	Post-PIVL: 28.1 ± 6.9;
	Final: 20.6 ± 5.8
Residual stenosis reduction	Post-PIVL absolute reduction: 48.5 percentage points (95% CI, 45.6 to 51.4);
	Final absolute reduction: 55.9 percentage points (95% CI, 53.1 to 58.8)
MLD, mm	Baseline: 1.3 ± 1.0;
	Post-PIVL: 3.8 ± 0.5;
	Final: 4.3 ± 0.6
Acute luminal gain after PIVL	2.5 ± 1.1 mm
Final residual stenosis ≤ 50%	20/20 lesions (100%)
Final residual stenosis ≤ 30%	18/20 lesions (90%)
Perforation, thrombus, distal embolization, abrupt closure, or no-reflow	0/20 lesions (0.0%)
Dissection	None: 15/20 (75.0%);
	Grade B: 3/20 (15.0%);
	Grade C: 2/20 (10.0%);
	Grade D or higher: 0/20 (0.0%)

Values are presented as n/N (%), mean ± standard deviation, or median (range), or as otherwise stated. Post-PIVL measurements were obtained immediately after PIVL and before adjunctive therapy. Final measurements were obtained after adjunctive therapy where performed. CI, confidence interval; DCB, drug-coated balloon; MLD, minimal luminal diameter; PIVL, pulsatile intravascular lithotripsy. * DCB platform not protocol-mandated or consistently captured. ** One balloon rupture prior to the rated lifespan of the catheter occurred.

**Table 3:** Follow-up, clinical outcomes, and safety.

Outcome	Value
Completed 30-day follow-up	9/9
Completed 6-month follow-up	9/9
Completed 12-month follow-up	5/9
Freedom from CD-TLR	100% at 30 days and 6 months; one event by 12 months
Duplex patency by PSVR (<50% restenosis)	20/20 lesions at 30 days; 19/20 lesions at 6 months
Primary patency, patient level	9/9 (100%) at 30 days; 8/9 (88.9%) at 6 months
Rutherford category improvement	8/9 (88.9%) at 30 days; 8/9 (88.9%) at 6 months among evaluable patients
ABI improvement among patients with PAD at baseline	All evaluable patients at 30 days and 6 months
EQ-5D-5L VAS score	47.5 ± 17.8 at screening (*n* = 6); 78.8 ± 17.1 at 30 days (*n* = 8); 73.6 ± 24.3 at 6 months (*n* = 8); 69.0 ± 16.7 at 12 months (*n* = 5)
Mean change in WIQ walking impairment score	30 days: +58.7; 6 months: +57.5; 12 months: +40.6
Mean change in WIQ walking distance score	30 days: +51.3; 6 months: +38.3; 12 months: +43.8
Mean change in WIQ walking speed score	30 days: +20.6; 6 months: +11.4; 12 months: +3.3
Mean change in WIQ stair-climbing score	30 days: +38.6; 6 months: +17.7; 12 months: +7.7
30-day MALE	0
30-day MAE	0
Procedure-related death	0
Serious adverse events during follow-up	15 events in five patients
Device-related serious adverse events	0
Procedure-related serious adverse events	One hematoma
Death during follow-up	One cardiac-failure death at 303 days; not device- or procedure-related
Device-related adverse events between 6 and 12 months	Three peripheral arterial restenosis events in two patients

Thirty-day and 6-month outcomes represent complete follow-up of the treated cohort. Twelve-month outcomes are exploratory because follow-up was available in five patients only. ABI, ankle-brachial index; CD-TLR, clinically driven target-lesion revascularization; MAE, major adverse event; MALE, major adverse limb event; PAD, peripheral arterial disease; VAS, visual analogue scale; WIQ, Walking Impairment Questionnaire.

**Table 4:** Analysis of populations, denominators, and outcome units.

Outcome Domain	Unit of Analysis	Denominator	Assessment Method
Device success	Patient	9 patients	Procedural case-report form
Balloon delivery, inflation, deflation, and retrieval	Device	15 balloons	Procedural case-report form
Technical success	Patient	9 patients	Procedural completion and angiographic result
Procedural success	Patient	9 patients	Device success + technical success + no procedural complication
Diameter stenosis	Lesion	20 lesions	Independent angiographic core laboratory
Minimal luminal diameter	Lesion	20 lesions	Independent angiographic core laboratory
Dissection, perforation, embolization, thrombus, abrupt closure, no-reflow	Lesion	20 lesions	Independent angiographic core laboratory
Duplex ultrasound patency	Lesion	20 lesions	Independent duplex ultrasound core laboratory; absence of ≥50% restenosis by PSVR criteria
Rutherford category	Patient	9 patients through 6 months	Clinical assessment
ABI	Patient	Patients with available paired ABI data	Physiological assessment
EQ-5D-5L and WIQ	Patient	Observed data by visit	Patient-reported outcome measures
CD-TLR, MALE, MAE, death, and serious adverse events	Patient	9 patients through 6 months; evaluable subset at 12 months	Independent clinical events committee

This table clarifies the unit of inference for each outcome domain to avoid the overstatement of precision in a first-in-human feasibility study in which several patients contributed more than one treated lesion. ABI, ankle-brachial index; CD-TLR, clinically driven target-lesion revascularization; MAE, major adverse event; MALE, major adverse limb event; WIQ, Walking Impairment Questionnaire.

## Data Availability

The data supporting the findings of this study are not publicly available because of confidentiality agreements with the study sponsor and restrictions relating to investigational device clinical trial data. Reasonable requests for de-identified data may be directed to the corresponding author and will be considered subject to sponsor approval, regulatory requirements, and applicable data-sharing agreements.

## Supplementary Materials

The following supporting information can be downloaded at: https://www.mdpi.com/article/10.3390/jcm15176589/s1, Table S1: TREND-based reporting checklist for this prospective, interventional, single-arm, non-randomized, first-in-human feasibility study [33]; Supplementary Information S1: Regarding Mixed Model Statistical Analysis; Supplementary Information S2: Regarding Sensitivity Analysis Excluding Common Femoral Artery Lesions; Table S2: Sensitivity analysis of angiographic outcomes according to lesion location.

## Abbreviations

ABI	ankle–brachial index
CD-TLR	clinically driven target lesion revascularization
DCB	drug-coated balloon
EH	electrohydraulic
IVL	intravascular lithotripsy
MAE	major adverse event
MALE	major adverse limb event
MLD	minimal luminal diameter
PAD	peripheral arterial disease
PARC	Peripheral Academic Research Consortium
PIVL	pulsatile intravascular lithotripsy
PSVR	peak systolic velocity ratio
PVI	peripheral vascular intervention
TLR	target lesion revascularization
WIQ	Walking Impairment Questionnaire
