# Supplementary material for: Pulsatile Intravascular Lithotripsy for Heavily Calcified Femoropopliteal Arterial Disease: First-in-Human Results from the POWER PAD I Feasibility Study
Source: J Clin Med. 2026 Aug 26;15(17):6589. doi: 10.3390/jcm15176589 (PMC13565774; doi:10.3390/jcm15176589)
Supplement: Supplementary file 1 [file jcm-15-06589-s001.zip › jcm-4493197-supplementary.pdf]

Journal of Clinical Medicine

MDPI

Supplementary Table S1. TREND-based reporting checklist for this prospective, single-arm, non-randomized feasibility study. TREND denotes Transparent Reporting of Evaluations with Nonrandomized Designs. The checklist has been adapted to the design of POWER PAD I, which was a prospective, interventional, single-arm, non-randomized, first-in-human feasibility study. Des Jarlais DC et al. Am J Public Health 2004;94:361–366.

Section	No.	Topic	POWER PAD I	Location
		**Title & Abstract**		
Title/Abstract	1	Study identified as non-randomized; design, intervention, and population named.	Title states "First-in-Human Feasibility Study." Abstract labels design as prospective, single-arm, two-center feasibility study. Intervention (PIVL) and population (calcified femoropopliteal PAD) explicit.	Title; Abstract
Title/Abstract	2	Structured abstract with design, methods, results, conclusions.	Four-part structured abstract: Background/Objectives, Methods, Results, Conclusions. Primary outcomes, key findings, and conclusion stated.	Abstract
		**Introduction**		
Background	3	Scientific background and rationale.	PAD epidemiology, impact of arterial calcification on endovascular outcomes, limitations of existing calcium-modification strategies, and preclinical PIVL data summarized.	Section 1
Hypotheses	4	Research questions and theoretical basis.	Feasibility objectives stated: safe delivery, acute luminal gain, and early safety of PIVL. Mechanism (water hammer effect converting pneumatic to hydraulic pulses) described.	Sections 1, 2.1
		**Methods**		
Participants	5	Inclusion and exclusion criteria.	Inclusion: age ≥ 18, RC 2–4, resting ABI ≤ 0.90 or exercise ABI ≤ 0.75, stenosis > 50%, vessel 4.0–6.5 mm, lesion ≤ 150 mm, at least moderate calcification (PARC). Exclusion: RC 5–6, graft/ISR, CTO > 40 mm, mild calcification only, aneurysm, thrombus.	Section 2.2
Participants	6	Recruitment method.	Consecutive prospective enrollment at two investigational sites following angiographic eligibility screening: 11 pre-screened; 9 enrolled and treated.	Sections 2.1, 3.1; Figure 1
Participants	**7**	Setting, location, and dates.	Royal Perth Hospital, Perth, Australia (*n* = 7), and Centro Medico Moderno, Santo Domingo, Dominican Republic (*n* = 2). Enrollment: July 2022–May 2023.	Sections 2.1, 3.1
Intervention	8	Intervention described in sufficient detail to allow replication.	Pulse IVL System: console, handle, and non-compliant balloon catheter over 0.014-inch guidewire. Pneumatic CO_2 pulses converted to hydraulic waves at—15 pulses/s; peak—50 atm; mean—6–8 atm. Minimum 30 s per lesion. Adjunctive DCB, embolic protection permitted at discretion.	Section 2.3
Objectives	9	Specific objectives and primary end points.	Primary performance: device success, technical success, procedural success (patient level). Primary safety: MALE at 30 days. Secondary: CD-TLR, residual stenosis < 30%, RC, ABI, EQ-5D-5L, WIQ, duplex patency.	Section 2.4
Outcomes	10	Primary and secondary outcomes and measurement methods.	Device success: delivery, inflation, deflation, retrieval without rupture. Technical success: access + completion + residual stenosis ≤ 50% by QCA. Safety (MALE, MAE) adjudicated by independent CEC. Angiography and duplex ultrasound by independent core laboratory.	Section 2.4

Sample Size	11	Sample size rationale.	No formal power calculation. Sample size (*n* = 9, 20 lesions) determined by first-in-human regulatory requirements and feasibility scope. Explicitly stated as exploratory.	Section 2.6
Assignment	12	Unit and method of assignment to intervention.	Single-arm study; all eligible participants received PIVL. No randomization or control group. Serial angiography (pre-PIVL, post-PIVL, post-adjunctive) provided internal comparisons.	Sections 2.1, 2.3
Blinding	13	Blinding of participants, operators, and outcome assessors.	Participants and operators not blinded (single-arm interventional study). Angiographic/duplex outcomes assessed by independent core laboratory; adverse events adjudicated by independent CEC, both blinded to clinical context.	Sections 2.1, 2.6
Unit of Analysis	14	Smallest unit used to evaluate intervention effects.	Three explicit units: (1) patient level—device/technical/procedural success, safety, PROs; (2) lesion level—angiographic outcomes, duplex patency; (3) device level—balloon delivery, rupture, retrieval. Summarized in Table 4.	Section 2.6; Table 4
Statistical Methods	15	Methods for primary and secondary analyses.	All analyses descriptives. Continuous: mean ± SD, median (range). Categorical: n/N (%). Confidence intervals at 95% level. Mixed model for angiographic change. No comparative tests performed. SAS v9.4.	Section 2.6
Statistical Methods	16	Additional and subgroup analyses.	No pre-specified subgroup analyses. 12-month outcomes pre-designated as exploratory (*n* = 5 of 9 patients). Within-patient clustering for lesion-level data acknowledged. No data imputation.	Section 2.6
		**Results**		
Participant Flow	17	Flow of participants through each study stage.	11 pre-screened; 9 enrolled and treated. All 9 completed 30-day and 6-month follow-up: 5 of 9 completed 12-month follow-up (1 died, 1 lost to follow-up, 2 protocol-complete at 6 months).	Section 3.1; Figure 1
Recruitment	18	Dates of recruitment and follow-up periods.	Enrollment: July 2022–May 2023. Follow-up: 30 days, 6 months (complete), 12 months (exploratory, 5/9 patients).	Section 3.1
Baseline Data	19	Baseline characteristics of the study cohort.	Median age 80 years, 5/9 male, Rutherford category 2–4. Total of 20 lesions: 95% severely calcified, 25% CTO. Mean baseline DS 76.5 ± 18.0%, MLD 1.3 ± 1.0 mm.	Section 3.2; Table 1
Numbers Analyzed	20	Numbers included in each analysis with reasons for exclusions.	All 9 treated patients included in all analyses (all-treated population). No exclusions. Lesion-level *n* = 20; device-level *n* = 15. Evaluable denominators stated at each time point. No imputation.	Sections 2.6, 3.1–3.5; Table 4
Outcomes	21	Summary of results for primary and secondary outcomes.	Device success 8/9 (88.9%; 95% CI, 51.8-99.7%) technical success 9/9 (100%); procedural success 8/9 (88.9%). DS: 76.5% → 28.1% (post-PIVL) ≥ 20.6% (final). MLD: 1.3 ≥ 3.8 ≥ 4.3 mm. Freedom from CD-TLR: 100% at 30d and 6mo. Zero MALE/MAE at 30d.	Sections 3.3-3.5; Tables 2–3
Ancillary Analyses	22	Other analyses performed.	Table 4 provides explicit denominator/unit-of-analysis mapping across all outcome domains. 12-month outcomes reported descriptively in the 5-patient evaluable subset.	Section 3.6; Table 4
Adverse Events	23	All-important adverse events.	0 MALE, 0 MAE, 0 procedure-related deaths at 30 days; 1 balloon rupture (post-therapy, auto shut-off, no sequelae); 1 procedure-related SAE (access-site hematoma); 15 SAEs in 5 patients (no device-related); 1 death at 303 days (cardiac failure, unrelated); 3 device-related AEs at 6–12 months (restenosis).	Section 3.5; Table 3
		**Discussion**		

Interpretation	24	Interpretation considering bias, internal validity, and limitations.	Findings interpreted as hypothesis-generating. Limitations: small sample (*n* = 9), no control group, DCB in 19/20 lesions limits attribution, no intravascular imaging, incomplete 12-month follow-up. Balloon rupture contextualized.	Section 4
Generalizability	25	External validity and applicability of findings.	Findings not generalizable to broader PAD populations given small *n*, two centers, no control, and anatomically complex cohort. Larger controlled trials with standardized protocols and longer follow-up required.	Section 4
**Overall Evidence**				
Overall Evidence	26	Interpretation in context of existing evidence.	PIVL results contextualized against atherectomy, scoring/cutting balloons, and electrohydraulic IVL (Disrupt PAD II/III). Mechanistic distinction of PIVL noted. Prior POWER PAD I research letter (Ref. 26) cited. Conclusion: first-in-human evidence supports further evaluation.	Sections 1, 4, 5

ABI, ankle–brachial index; AE, adverse event; CEC, clinical events committee; CD-TLR, clinically driven target-lesion revascularization; CTO, chronic total occlusion; DCB, drug-coated balloon; DS, diameter stenosis; ISR, in-stent restenosis; MAE, major adverse event; MALE, major adverse limb event; MLD, minimal luminal diameter; PAD, peripheral arterial disease; PARC, Peripheral Academic Research Consortium; PIVL, pulsatile intravascular lithotripsy; PRO, patient-reported outcome; QCA, quantitative coronary angiography; RC, Rutherford category; SAE, serious adverse event; SD, standard deviation; WIQ, Walking Impairment Questionnaire.

Supplementary Information S1. Regarding Mixed Model Statistical Analysis

The data used in the model has one row per lesion per time point for each subject. In this data, the subject is identified by a unique ID and lesion segment is also identified by a unique ID. The time point has a value of 1 for the post-IVL assessment and 2 for the post-adjunctive (final) assessment. The outcome variable is the change from the pre-IVL stenosis (i.e. post-IVL minus pre-IVL for time point 1, and post-adjunctive minus pre-IVL for time point 2). The model includes the time point as a categorical variable and the pre-IVL stenosis as a continuous covariate. The subject ID, nesting the segment ID, is used as the repeated measures unit and included in the SAS code. Due to the limited amount of data and the exploratory nature of the analysis, the SAS default method of estimating variance components was used in the final analysis.

As the SAP did not specify a model for analysis of change in stenosis, and the sample size was limited, the outcome from the model and corresponding 95% confidence limits were considered exploratory and post-hoc.

Supplementary Information S2: Sensitivity Analysis Excluding Common Femoral Artery Lesions

Three of the 20 treated lesions were subsequently classified by independent core laboratory adjudication as being in the common femoral artery (CFA). Because protocol eligibility was limited to lesions of the superficial femoral and popliteal arteries, a sensitivity analysis was performed excluding these three lesions.

Among the 17 non-CFA lesions, mean diameter stenosis decreased from 74.6% at baseline to 28.9% immediately following PIVL, and mean minimum lumen diameter (MLD) increased from 1.4 mm to 3.7 mm. Following adjunctive treatment, mean diameter stenosis was 20.8% and mean MLD was 4.3 mm. Thus, exclusion of the three CFA lesions did not materially alter the principal acute angiographic findings.

For descriptive comparison, outcomes for the three CFA lesions are also presented in Supplementary Table S2. Because of the small number of CFA lesions, these data are descriptive and no formal between-group statistical comparisons were performed.

Supplementary Table S2. Sensitivity analysis of angiographic outcomes according to lesion location

	Pre-treatment Stenosis (%) / MLD (mm)	Post-IVL Stenosis (%) / MLD (mm)	Final Stenosis (%) / MLD (mm)
Non-CFA (SFA/pop-liteal), n-17	74.6 / 1.4	28.9 / 3.7	20.8 / 4.3
CFA, n=3	87.6 / 1.2	28.1 / 4.0	19.8 / 4.4
